# In silico design of a multiantigenic and multiepitope chimeric protein as a vaccine candidate against *Renibacterium salmoninarum*

**DOI:** 10.3389/fcimb.2026.1814020

**Published:** 2026-06-05

**Authors:** Jeffrey Araneda, Patricio A. Flores-Herrera, Waldo Acevedo, Sergio H. Marshall, Fernando A. Gómez

**Affiliations:** 1 Laboratorio de Genética e Inmunología Molecular, Instituto de Biología, Facultad de Ciencias, Pontificia Universidad Católica de Valparaíso, Valparaíso, Chile; 2 Centro GEMA- Genómica, Ecología y Medio Ambiente, Universidad Mayor, Santiago, Chile; 3 Laboratorio de Química Biológica, Instituto de Química, Facultad de Ciencias, Pontificia Universidad Católica de Valparaíso, Valparaíso, Chile; 4 Center for Interdisciplinary Research in Biomedicine, Biotechnology and Well-Being (CID3B), Pontificia Universidad Católica de Valparaíso, Valparaíso, Chile

**Keywords:** bacterial kidney disease, chimeric protein, immunoinformatics, *in silico* vaccine design, multiepitope vaccine, *Renibacterium salmoninarum*, reverse vaccinology, salmon aquaculture

## Abstract

Bacterial kidney disease (BKD), caused by the Gram-positive intracellular bacterium *Renibacterium salmoninarum*, remains a major challenge for salmon aquaculture, particularly in Chile, where the lack of an effective vaccine has led to sustained antibiotic use. In this study, we applied an integrative *in silico* approach combining comparative genomics, reverse vaccinology, immunoinformatics, structural modeling, molecular docking, and molecular dynamics to design a multiantigenic and multiepitope chimeric protein as a vaccine candidate. Comparative analysis of three genomes revealed a highly conserved proteome, supporting the identification of shared immunogenic targets. From proteins associated with virulence, secretion systems, iron acquisition, chaperones, ribosomal functions, magnesium transport, and carbohydrate uptake, 17 proteins were selected after antigenicity and localization screening. Epitope prediction initially identified 154 candidates; after filtering for antigenicity, toxicity, and host homology, 60 epitopes grouped into 26 antigenic regions derived from 15 proteins were retained. Structural models were evaluated using QMEAN and Ramachandran analysis, and docking against Atlantic salmon MHC class I and II alleles identified the best candidates, with HADDOCK scores ranging from −71.2 to −154.6 (MHC I) and −116.5 to −152.0 (MHC II). Twelve antigenic regions were assembled into a 439-amino-acid chimeric protein using GSGSGS linkers. The construct showed a predicted molecular weight of 43.7 kDa, pI of 9.56, aliphatic index of 87.97, moderate solubility (0.43), and no predicted toxicity. Molecular dynamics (100 ns) supported structural stability, with RMSD convergence at ~85 ns. Surface analysis identified exposed antigenic regions mainly from DnaK, HmuU, Tpl, and DacB. This approach enabled the rational design of a structurally stable and immunologically promising candidate, providing a basis for future experimental validation.

## Introduction

Salmon aquaculture has become one of the most relevant productive sectors in Chile, positioning the country as the second largest salmon producer worldwide after Norway. This growth has been accompanied by significant economic benefits; however, it has also been associated with an increased vulnerability to infectious diseases, which continue to pose major challenges to the sustainability of the industry (Wolf et al., 2014; SERNAPESCA, 2024a). Among these, bacterial infections have been particularly problematic, largely due to their persistence, capacity for horizontal and vertical transmission, and the limited effectiveness of available preventive measures. Therefore, disease management in salmon farming has relied heavily on the use of antibiotics, raising growing concerns regarding environmental contamination and the emergence of antimicrobial resistance (Oceana, 2018).

Bacterial kidney disease (BKD) is one of the most important bacterial diseases affecting salmon aquaculture worldwide and represents a major concern in Chilean production systems. The disease is caused by the Gram-positive, slow growing, obligate intracellular bacterium *R. salmoninarum* and primarily affects economically relevant salmonid species such as Salmo salar, Oncorhynchus mykiss, and *Oncorhynchus kisutch* (Bruno, 2015). BKD is characterized by a chronic granulomatous infection, with clinical signs including skin darkening, abdominal distension, renal hypertrophy, and the presence of white nodules in internal organs, often leading to high mortality rates and significant economic losses (Bayliss et al., 2018). Official sanitary data from 2024 indicate that in Chile BKD accounted for 9.2% of pathogen-associated mortalities in Atlantic salmon (*Salmo salar*), 12.1% in coho salmon (*Oncorhynchus kisutch*), and 8.4% in rainbow trout (*Oncorhynchus mykiss*), based on confirmed laboratory diagnoses reported by SERNAPESCA. Although its contribution to total mortality is moderate, BKD continues to exert a disproportionate impact on disease management, as renibacteriosis represented 51.5% of antimicrobial use by disease in freshwater production stages during 2024, reinforcing its relevance as a target for alternative preventive strategies (SERNAPESCA, 2024a; SERNAPESCA, 2024b).

In Chile, *R. salmoninarum* was first isolated in 1986, and subsequent phylogenetic analyses have suggested that the pathogen was introduced on multiple occasions through the importation of salmonid stocks from North America, Europe, and Japan during different phases of the expansion of the salmon farming industry (Sanders and Barros, 1986). Both vertical and horizontal transmission routes have been documented, further complicating control strategies and contributing to the persistence of the disease within and between production cycles (Fryer and Sanders, 1981; Bullock and Leek, 1986). At the molecular level, the virulence of *R. salmoninarum* has been associated with a complex interplay of secreted enzymes, toxins, iron acquisition systems, and surface-associated proteins, including the major soluble antigen p57, which has been described as a key immunosuppressive and virulence factor (Bruno, 2015).

Despite extensive efforts to control BKD, current preventive strategies remain insufficient. The only commercially available vaccine, Renogen^®^, is based on proteins derived from Arthrobacter sp., a phylogenetically related organism, and has shown limited protective efficacy under field conditions (Elliott et al., 2014). As a result, antibiotic treatments remain the primary control measure, particularly during outbreaks. In Chile, antibiotic usage in salmon aquaculture is considerably higher than in other major producing countries, such as Norway, a situation that has raised serious environmental and public health concerns (Oceana, 2018). Notably, several studies have reported the presence of antibiotic resistance determinants in marine bacteria from aquaculture-impacted environments and their potential horizontal transfer to human pathogens (Aedo et al., 2014; Tomova et al., 2015, Tomova et al., 2018).

Advances in bioinformatics, genomics, and molecular biology have enabled the development of alternative vaccine design strategies that overcome some of the limitations of traditional approaches. Reverse vaccinology, immunoinformatics, and structural vaccinology allow the systematic screening of pathogen genomes and proteomes to identify immunogenic proteins and epitopes with high potential to induce protective immune responses (He et al., 2010; Seib et al., 2012). These methodologies have been successfully applied to the design of vaccine candidates against a wide range of infectious agents, including viral, parasitic, and bacterial pathogens of medical and veterinary importance (Negahdaripour et al., 2017; Vakili et al., 2018; Murphy et al., 2019).

The immune system of teleost fish is considered well developed and shares several functional features with that of higher vertebrates, including both innate and adaptive components (Uribe et al., 2011). However, adaptive immune memory in teleosts has been primarily associated with B lymphocytes and antibody-mediated responses, highlighting the importance of antigen accessibility and epitope selection in vaccine design (Morrison and Nowak, 2002; Ye et al., 2013; Gloanec et al., 2025). In this context, surface-exposed, secreted, and virulence-associated proteins, as well as conserved immunogenic epitopes, represent particularly attractive targets for the rational development of vaccines against BKD.

In the present study, an integrative *in silico* approach combining reverse vaccinology, immunoinformatics, and structural vaccinology was applied to the genome and proteome of *R. salmoninarum* to identify immunogenic proteins from diverse functional subsystems. Through comparative genomic and proteomic analyses, conserved antigenic targets were selected and systematically evaluated based on antigenicity, subcellular localization, epitope content, toxicity, and host homology. The most promising antigenic regions were structurally modeled and assessed for their interaction with salmonid MHC class I and II molecules, allowing the rational assembly of a multiantigenic and multiepitope chimeric protein. This strategy highlights the potential of integrative *in silico* methodologies to support the design of novel vaccine candidates against BKD and provides a robust framework for future experimental validation aimed at improving disease control and reducing antibiotic dependence in salmon aquaculture.

## Materials and methods

### *In silico* characterization of the *R. salmoninarum* genome and proteome

The available genomes of *Renibacterium salmoninarum* strains ATCC 33209 (GenBank: CP000910), H-2 (GenBank: CP029236), and DJ2R (GenBank: CP029237) were retrieved from the NCBI database. Whole-genome alignment was performed using Mauve (v2.3.1) to assess genome conservation and identify potential speciation events or horizontal gene transfer among strains (Darling et al., 2004). Given the high level of genomic conservation observed among the three strains, subsequent analyses focused on conserved proteomic features rather than strain-specific variation. For each genome, automated proteomic annotation was conducted using RAST (Rapid Annotation using Subsystem Technology), version 4, to ensure a consistent functional classification across all analyzed genomes (Aziz et al., 2008). RAST was selected because it provides access to the SEED Subsystems framework, which organizes genes into hierarchical functional categories and facilitates comparative analyses of metabolic pathways, virulence-associated factors, and secretion system components. This unified annotation approach minimized annotation bias and ensured that all genomes were analyzed under identical functional criteria. This annotation was complemented with UniProt-based searches to identify secretion systems and previously described proteins (O’Farrell and Strom, 1999; Brynildsrud et al., 2016). In addition, proteins associated with virulence and antibiotic resistance conserved in *R. salmoninarum* were included based on previous *in silico* analyses performed in phylogenetically related species, particularly within the genus *Mycobacterium* (Bethke et al., 2018). This combined approach was used to generate the initial dataset of candidate proteins for downstream antigenicity and epitope prediction analyses.

### Selection of immunogenic proteins

From the proteomic dataset obtained from RAST annotation and complementary UniProt-based searches, proteins were selected based on their potential to induce an immune response. Priority was given to proteins associated with membrane localization, motility, adhesion, secretion systems, virulence, chaperones, and ribosomal functions, as these have been previously described as relevant immunogenic targets. All selected proteins were evaluated for antigenicity using the VaxiJen webserver, and those with scores above 0.5 were considered probably antigenic, following the recommended threshold (Doytchinova and Flower, 2007). Given that immune memory in teleost fish has been mainly associated with B lymphocytes and antibody production (Morrison and Nowak, 2002), special emphasis was placed on membrane-bound and secreted proteins due to their higher accessibility to the immune system.

Subcellular localization was predicted using the Protter, Gpos-mPLoc, and PSORTb webservers to support this selection (Omasits et al., 2014; Shen and Chou, 2009; Yu et al., 2010). Proteins consistently predicted as extracellular, membrane-associated, or secreted were prioritized. In addition to surface-associated proteins, chaperones and ribosomal proteins were also included because of their reported immunomodulatory properties (Neckers and Tatu, 2008; Johnson et al., 2017). Although intracellular proteins are not directly exposed on the pathogen surface, they may become accessible to the host immune system through antigen processing and presentation pathways. In intracellular pathogens such as *R. salmoninarum*, these proteins can be presented via MHC molecules, contributing to T-cell–mediated immune responses. In addition, several intracellular proteins, including chaperones and ribosomal proteins, have been reported to exhibit immunogenic and immunomodulatory properties, supporting their inclusion as potential vaccine targets.

Following these criteria, proteins meeting both antigenicity and localization requirements were retained for subsequent epitope prediction analyses, resulting in a final set of candidate immunogenic proteins.

### Epitope prediction

Selected proteins were subjected to epitope prediction analyses to identify antigenic regions with the potential to stimulate B- and T-cell responses. B-cell epitopes were predicted using the IMED and BcePred webservers, which evaluate physicochemical properties such as hydrophilicity, flexibility, accessibility, polarity, and surface exposure (Kolaskar and Tongaonkar, 1990; Saha and Raghava, 2006). Default parameters were used for both tools, and predicted epitopes were initially retained to allow broad identification of candidate antigenic determinants. In parallel, T-cell epitope prediction was performed using the IEDB CD4+ prediction tool, based on the 7-allele reference method described by Paul et al. (2015). Predicted epitopes were selected according to their binding potential to representative MHC class II alleles, following the scoring criteria implemented in the IEDB platform.

For membrane-associated proteins, only epitopes located in extracellular regions were considered, based on topology predictions obtained with Protter (Omasits et al., 2014). All predicted epitopes were subsequently evaluated for antigenicity using the VaxiJen webserver, and those with higher scores were prioritized for further analysis (Doytchinova and Flower, 2007).

To ensure safety, toxicity of the predicted epitopes was assessed using the ToxinPred webserver, and peptides predicted as toxic were excluded (Gupta et al., 2013). In addition, to minimize the risk of host cross-reactivity, the proteins containing the selected epitopes were aligned against teleost host proteomes (*Salmo salar*, *Oncorhynchus mykiss*, and *Oncorhynchus kisutch*) using BLASTp. Only epitopes located in non-conserved regions were retained after this filtering step (Morrison and Nowak, 2002). This sequential filtering process resulted in an initial set of 154 predicted epitopes, which were reduced to 60 epitopes grouped into 26 antigenic regions derived from 15 proteins, and subsequently used for structural modeling and downstream analyses.

### Three-dimensional structure modeling of epitopes

For the selected antigenic regions, three-dimensional structural models were generated to preserve their conformational characteristics. Protein structure prediction was performed using the Phyre2, RaptorX, and I-TASSER webservers, as well as Modeller 9.22, which combine homology detection, threading, and comparative modeling approaches to generate structural models (Sali and Blundell, 1993; Källberg et al., 2014; Kelley et al., 2009; Yang et al., 2015). For each protein, multiple structural models were generated using the different prediction platforms. Model quality was evaluated using the QMEAN server, including QMEANDisCo and QMEANBrane scoring functions, to assess both global and local reliability (Studer et al., 2014; Waterhouse et al., 2018). In addition, stereochemical quality was evaluated using Ramachandran plot analysis with the RAMPAGE webserver. Models showing the highest QMEAN scores and the best distribution of residues in energetically favored regions were selected for subsequent analyses. Template selection and model construction were performed based on sequence similarity and structural coverage as provided by each prediction platform. This multi-tool strategy allowed cross-validation of predicted structures, reducing potential biases associated with any single modeling method and ensuring consistency in the structural representation of antigenic regions.

Although recent deep learning-based methods such as AlphaFold have significantly improved protein structure prediction accuracy, the objective of this study was not to obtain a single high-confidence structural model, but rather to preserve and compare the conformational consistency of selected antigenic regions for downstream analyses, including epitope selection and molecular docking. In this context, the use of multiple complementary modeling approaches allowed cross-validation of predicted structures and identification of conserved structural features across independently generated models. This strategy was therefore considered appropriate for the objectives of this study.

The selected models, which showed high structural quality based on QMEAN scores and Ramachandran analysis, were subsequently used for molecular docking analyses against Atlantic salmon MHC class I and II alleles.

### Protein–ligand docking analysis

Based on the selected structural models, the complete secondary structures encompassing each antigenic region were used for molecular docking analyses in order to preserve their native three-dimensional conformation. Protein–ligand docking was performed between the selected antigenic regions and Atlantic salmon (*Salmo salar*) MHC class I (allele UBA*0201)* and MHC class II *(alleles DAA*0201–DAB*0201), which have been associated with increased resistance to infectious diseases in salmonids (Kjøglum et al., 2006). The three-dimensional structures of these MHC molecules were obtained from previously published models (Cárdenas et al., 2010). Docking simulations were carried out using the HADDOCK 2.2 webserver, which allows flexible protein–protein docking and ranks predicted complexes based on a scoring function that integrates intermolecular energies and surface accessibility parameters (Dominguez et al., 2003; De Vries et al., 2007). In the absence of experimentally validated interaction interfaces, no predefined active or passive residues were assigned, and docking was performed using default parameters. For each interaction, docking solutions with the lowest HADDOCK scores, indicative of more favorable binding energies, were considered for subsequent selection of antigenic regions.

### Rational design of the chimeric construct

The chimeric protein was designed by combining the antigenic regions that showed the highest antigenicity scores and the most favorable immunomodulatory potential, selected from different functional subsystems of *R. salmoninarum*. Priority was given to antigenic regions that also exhibited the strongest interactions with Atlantic salmon MHC class I and II alleles in the docking analyses. The selected epitopes were linked sequentially using the flexible amino acid linker GSGSGS, which has been commonly employed to preserve epitope independence and structural flexibility in multiepitope vaccine constructs (Yang et al., 2015; Solanki & Tiwari, 2018). This strategy allowed the generation of a single multiantigenic and multiepitope chimeric protein, designed to maximize immune recognition while maintaining structural integrity.

### *Ab initio* modeling, refinement, and quality assessment of the chimeric protein

Due to the absence of close homologous templates, ab initio modeling of the chimeric protein was performed using the Robetta webserver, which applies *de novo* structure prediction and fragment assembly approaches for protein modeling (Kim et al., 2004). A total of five structural models were generated, and the model showing the best overall quality and preservation of the original three-dimensional conformation of each antigenic region was selected. Structural features and epitope integrity of the selected model were evaluated using the PDBsum database (Laskowski et al., 2018). Overall and local model quality were assessed using QMEAN, including the QMEANDisCo scoring function, which estimates model reliability based on statistical potentials derived from experimentally resolved protein structures (Studer et al., 2014; Waterhouse et al., 2018).

The quality’s model was tested using the Ramachandran plot analysis, ProsaWeb analysis (https://prosa.services.came.sbg.ac.at/prosa.php) and the DOPE energies analysis from the modeller’s evaluate script, v9.23. The chimera quality was got before and after refinement. *The* antigenic evaluation was performed by using the EPCES webserver for antigenic prediction in protein’s surface models (http://sysbio.unl.edu/EPCES/). Finally, the physicochemical characteristics were obtained using the ProtParam (http://web.expasy.org/protparam) and Protein-Sol (https://protein-sol.manchester.ac.uk/) webservers. Finally, its toxicity was discarded with the ToxinPred protein scanning (https://webs.iiitd.edu.in/raghava/toxinpred/protein.php).

### Molecular dynamics simulation

A molecular dynamics (MD) simulation of the Chimeric Protein was carried out using NAMD (version 2.3) and following standard procedures described in the NAMD tutorial provided by the Theoretical and Computational Biophysics Group. The initial structure was prepared using VMD, and the corresponding protein structure file (PSF) was generated. The system was modeled using the CHARMM27 force field with CMAP corrections, which improves the representation of protein backbone conformations. The protein was solvated in an explicit water box using the TIP3P water model, with a standard buffer distance. Periodic boundary conditions were applied in all three spatial dimensions to mimic an infinite bulk solvent environment. To simulate physiological ionic conditions, the system was neutralized with Na^+^ and Cl^-^ ions to achieve a final salt concentration of 0.15 M. Ions were placed in energetically favorable positions within the solvent to ensure system stability. Long-range electrostatic interactions were calculated using the Particle Mesh Ewald (PME) method, while short-range nonbonded interactions were measured with a cutoff of 12 Å and a switching function applied from 10 Å. A pair list distance of 14 Å was used.

Prior to production, the system underwent energy minimization for 20,000 steps. This was followed by equilibration under constant temperature and pressure conditions. Temperature was maintained at 310 K using Langevin dynamics with a damping coefficient of 1 ps^-^¹, while pressure was controlled at 1 atm. All bonds involving hydrogen atoms were constrained, enabling the use of a 2-fs integration time step, and the equations of motion were integrated using a Verlet algorithm.

A production simulation was then performed in the NPT ensemble (constant number of particles, pressure, and temperature) for a total of 100 ns. Trajectories were recorded at regular intervals for subsequent structural and dynamical analyses. The structural stability and flexibility of the system were analyzed by computing the root mean square deviation (RMSD) and root mean square fluctuation (RMSF) from the molecular dynamics trajectories using VMD.

### Prediction of physicochemical properties, antigenicity, and toxicity of the chimeric protein

The immunogenic and antigenic potential of the designed chimeric protein was evaluated using the VaxiJen, IMED, IEDB CD4+ prediction tool, and BcePred webservers, following the same criteria applied during epitope selection (Doytchinova and Flower, 2007; Kolaskar and Tongaonkar, 1990; Saha and Raghava, 2006; Paul et al., 2015). These analyses allowed the assessment of the overall antigenicity and the capacity of the construct to stimulate B- and T-cell–mediated immune responses. Physicochemical properties of the chimeric protein, including amino acid length, molecular weight, theoretical isoelectric point, hydrophobicity, aliphatic index, and estimated half-life, were calculated using the ProtParam webserver (Wilkins et al., 1999). Protein solubility under aqueous conditions was predicted using the Protein-Sol webserver, providing an estimate of the construct’s solubility behavior in solution (Hebditch et al., 2017). Finally, the potential toxicity of the chimeric protein was evaluated using the ToxinPred webserver to exclude toxic motifs and ensure compatibility with eukaryotic systems (Gupta et al., 2013).

All web-based tools were used through their publicly available servers under default settings. Due to the dynamic nature of web platforms, specific version numbers were not always available; however, all analyses were conducted between 2023 – 2025, ensuring consistency across the study.

## Results

### Genomic conservation and proteomic annotation of *R. salmoninarum*

Comparative genomic analysis of the three *Renibacterium salmoninarum* strains (ATCC 33209, H-2, and DJ2R) revealed a high level of conservation across genomes. Whole-genome alignment using Mauve showed a single collinear block spanning the entire genome, with only minor localized variations, indicating strong genomic stability among strains (**Supplementary Figure 1**). Proteomic annotation identified approximately 3,700 coding sequences per genome, which were organized into 260 functional subsystems showing high similarity among strains (**Supplementary Table 1**). This conserved proteomic landscape supported the use of a shared dataset for downstream analyses. Proteins associated with virulence, secretion systems, iron acquisition, cell wall and capsule, and ribosomal and chaperone functions were identified within this dataset (**Supplementary Tables 2**, **3**). Homology analysis further confirmed conservation of several of these proteins across phylogenetically related bacterial species (**Supplementary Table 4**).

From the initial proteomic dataset, antigenicity screening using VaxiJen identified 27 proteins with scores above the 0.5 threshold (**Supplementary Table 5**). Subsequent subcellular localization analysis classified these proteins into extracellular, membrane-associated, and intracellular categories (**Supplementary Table 6**). Based on antigenicity, localization, and functional relevance, this filtering process resulted in a final set of 17 proteins selected for epitope prediction analyses.

### Selection of immunogenic proteins, antigenic regions, and structural evaluation

Proteomic datasets obtained from RAST, UniProt, and previously published *in silico* analyses were used to identify candidate immunogenic proteins from *R. salmoninarum*. Proteins associated with diverse functional subsystems, including virulence, secretion systems, iron acquisition and metabolism, chaperones, ribosomal functions, magnesium acquisition, and carbohydrate transport, were prioritized for further analysis (**Supplementary Table 5**). From an initial proteomic dataset comprising approximately 3,700 coding sequences per genome (3,715–3,719), antigenicity screening using the VaxiJen server identified 27 proteins with scores above the recommended threshold of 0.5 (**Supplementary Table 5**). Subsequent subcellular localization predictions identified proteins predicted to be extracellular, membrane-associated, or secreted as preferential targets, given their higher accessibility to the host immune system. Chaperones and ribosomal proteins were also retained due to their reported immunomodulatory properties (**Supplementary Table 6**). This multi-step filtering process resulted in a final set of 17 proteins selected for epitope prediction analyses (**Table 1**).

**Table 1:** Proteins selected for epitope and antigenic region identification.

Subsystem	Protein name	UniProt accession	VaxiJen score
Chaperones	HSP70 (DnaK)	A9WQR3	0.6346
	HSP40 (DnaJ)	A9WQT0	0.8160
Virulence	p57	Q9R3F0	0.6465
	D-alanyl-D-alanine carboxypeptidase	A9WUR8	0.5142
	Tyrosine phenol-lyase	A9WN89	0.5116
Secretion systems	Type IV secretion system protein	A9WP76	0.5557
	TadE family protein	A9WN91	0.5819
	Type IV secretion system protein	A9WP76	0.5557
Iron acquisition and metabolism	Heme ABC transporter, cell surface heme and hemoprotein receptor HmuT	A9WRJ5	0.7008
	Hemin transport system permease protein, HmuU1	A9WRJ2	0.6003
	Heme transport associated protein	A9WRJ6	0.5903
	Transport system permease protein	A9WMV7	0.5518
Ribosomal proteins	SSU ribosomal protein S12p	A9WSW8	0.8617
	SSU ribosomal protein S7p	A9WSW7	0.7114
	LSU ribosomal protein L35	A9WQB0	0.7438
Magnesium acquisition	Mg²^+^ transport ATPase protein C	A9WQM5	0.5913
Sugar transport	Sugar ABC transporter permease	A9WUD1	0.5533

Proteins were selected mainly from secretion, virulence, chaperone, iron acquisition and metabolism, ribosomal protein, Mg²^+^ acquisition, and sugar transport subsystems. Selection criteria included higher predicted antigenicity scores (VaxiJen) and/or membrane or extracellular localization, with the aim of representing different functional subsystems. In total, 17 proteins were selected for antigenic region identification.

Epitope prediction using IMED, BcePred, and the IEDB CD4+ prediction tool yielded an initial pool of 154 putative epitopes corresponding to 69 antigenic regions (**Supplementary Table 7**). Following antigenicity scoring and consolidation of overlapping predictions, 60 epitopes grouped into 26 antigenic regions, derived from 15 proteins belonging to different functional subsystems, were selected as the most promising candidates (**Table 2**). All selected epitopes were predicted to be non-toxic (**Table 3**), and none of the selected antigenic regions were conserved in the proteomes of *S. salar*, *O. mykiss*, or *O. kisutch*, minimizing the risk of host cross-reactivity (**Supplementary Table 8**).

**Table 2:** Selected antigenic regions.

Protein name	UniProt accession	BcePred epitope	Position	VaxiJen score	IEDB epitope	Position	VaxiJen score	IMED epitope	Position	VaxiJen score
Tyrosine phenol-lyase	A9WN89	AGVFTSSSSGGCSRSLQTGIDGGSGQKRRDRHDDPAC	97–131	1.4517	–	–	–	SSSGCSR	103–109	2.7791
TadE family protein	A9WN91	–	–	–	ITTLKITVRAPMPII	96–110	0.9234	TTLKITVRAPMPIIGLIGL	97–115	0.6010
Type II secretion system protein	A9WPT5	IVHPPINFLIP	74–85	1.6941	HPPINFLIPAGISI	76–90	0.9747	RSATVTLGAIVHPPINFLIPAGISISA	65–92	0.8045
SSU ribosomal protein S12p	A9WSW8	NQLVRKGRSPKVAKTKAPALKNPMRRGVCTVRYTTTPKPKPNSALRKVARRVL	5–57	0.7049	GRSPKVAKTKAPALK	11–25	1.1829	RSPKVAKTKAP	12–22	0.6907
SSU ribosomal protein S12p	A9WSW8	RGCVTRVYT	31–39	1.9531	–	–	–	RRGVCTRVYT	30–39	1.6390
D-alanyl-D-alanine carboxypeptidase	A9WUR8	TQGPDANVRRA	377–387	1.9976	QVMRLMTQGPDANVR	371–385	1.0967	DPGDVAVGEIAPIFPLAVNSARADVKSAG	213–242	1.1057
D-alanyl-D-alanine carboxypeptidase	A9WUR8	–	–	–	DVAVGEIAPIFPLAV	216–230	1.3446	–	–	–
Sugar ABC transporter	A9WUD1	–	–	–	FFFMFAFDGVNIKIF	171–185	1.8260	AFDGVIN	176–182	0.3627
Mg²^+^ transport ATPase protein C	A9WQM5	LPEERLRSVQSDSDATP	174–190	1.4055	EFRLRSVQSDSDATP	176–190	1.5310	TPGEVRIAAEL	189–199	0.8803
Mg²^+^ transport ATPase protein C	A9WQM5	AELTAHERDDRQLEAAVSRLSLEPRV	199–222	1.1776	AAVSRLSLEPRVTSV	211–225	0.9762	LEAAVSRLSLEPRVTSVRW	209–227	1.1867
p57	Q9R3F0	SGDNNTYGG	344–352	2.0829	–	–	–	–	–	–
p57	Q9R3F0	–	–	–	GTVVKVDGSNLFGAS	166–180	0.5563	GGGTVVKVDG	164–173	1.9929
p57	Q9R3F0	–	–	–	FLYLRGSGVYYLFG	251–265	0.7256	GGSGVYYLF	256–264	1.2794
Hemin-binding periplasmic protein (HmuT)	A9WRJ5	ASHDRGGDVD	87–96	2.3752	–	–	–	GGDVQV	93–99	2.1329
Hemin-binding periplasmic protein (HmuT)	A9WRJ5	TSPNGQSVNAE	146–156	1.7749	–	–	–	VNAESILALKPSVVIT	153–168	0.8112
Hemin-binding periplasmic protein (HmuT)	A9WRJ5	–	–	–	KVSAQKLRMAFLYLR	241–255	1.5472	KLRMAFLYL	246–254	1.9845
Hemin-binding periplasmic protein (HmuU.1)	A9WRJ2	LLFSTAGQSSAGQA	126–139	1.206	IVAPLLFSTAGQSS	121–135	0.9395	AAFGASAAVAPLLFS	113–129	0.8149
dnaJ	A9WQT0	EVLSDPQKRQVYDTTNGENTQSGGSGF	52–79	1.4018	–	–	–	–	–	–
dnaJ	A9WQT0	VEMRVNRDAKFDREGDDLHA	246–265	1.4197	VEMRVNRDAKFDREG	246–260	1.1884	–	–	–
Ferric enterobactin transport system	A9WMV7	TVRLGVPSVQLKP	3–15	1.3871	MTTVRLGVPSVQLKPRAVLISAVLFVLALMAFHVAYGG	6–40	0.9078	VRLGPVSFQLKPRAVLISAVLFVLVLALMAFHVAYGGTALPYGKVFVALLG	4–54	0.5525
Type IV secretion system	A9WP76	TLVVVTLWLVKVVRKR	14–30	0.3496	IGATLVVVTLWLVV	11–25	1.0846	SSTAAVLIGATLVVVTLWLVVKV	4–27	0.5480
HSP70 (DnaK)	A9WQR3	EDELLLVFDLGGGTFDVSLLEVG	162–182	0.7545	KEDELLLVFDLGGGT	161–175	1.0489	TFDVSLLEV	175–183	1.3415
HSP70 (DnaK)	A9WQR3	GPLDIHVLDVTF	148–160	1.2185	DIHVLDVTFANPSVT	151–165	1.2243	PLDIHVLDVTFANPSVTI	149–166	1.2727
Heme transport associated protein	A9WRJ6	SMQPVPLTLNVP	642–653	0.8978	ASSPRISMQPVPLTL	636–650	1.2957	ISMQPVPLTLNVPTSGQTAVFRVEASASPA PSVQW	641–677	1.0301
Heme transport associated protein	A9WRJ6	VTTEKAPEPQLPIDPTATSRPGST	731–754	1.0469	–	–	–	PEPQLPID	737–744	1.2928
Heme transport associated protein	A9WRJ6	–	–	–	LNLTFSNPQLSIDSA	826–840	1.2520	FSNPQLSID	830–838	0.8122

Sixty epitopes derived from 15 proteins were selected and grouped into 26 antigenic regions based on epitope overlap among prediction servers.

**Table 3:** Toxicity prediction of epitopes.

Protein name	UniProt accession	BcePred epitope	Prediction	IEDB epitope	Prediction	IMED epitope	Prediction
Tyrosine phenol-lyase	A9WN89	AGVFTSSSSGGCSRSLQTGIDGGSGQKRRDRHDDPAC	Non-toxin	–	–	SSSGCSR	Non-toxin
TadE family protein	A9WN91	–	–	ITTLKITVRAPMPII	Non-toxin	TTLKITVRAPMPIIGL	Non-toxin
Hypothetical protein	A9WPT5	IVHPPINFLIP	Non-toxin	HPPINFLIPAGISI	Non-toxin	RSATVTLGAIVHPPINFLIPAGISISA	Non-toxin
SSU ribosomal protein S12p	A9WSW8	NQLVRKGRSPKVAKTKAPALKNPMRRGVCTVRYTTTPKPKPNSALRKVARRVL	Non-toxin	GRSPKVAKTKAPALK	Non-toxin	RSPKVAKTKAP	Non-toxin
SSU ribosomal protein S12p	A9WSW8	RGCVTRVYT	Non-toxin	–	–	RRGVCTRVYT	Non-toxin
D-alanyl-D-alanine carboxypeptidase	A9WUR8	TQGPDANVRRA	Non-toxin	QVMRLMTQGPDANVR	Non-toxin	DPGDVAVGEIAPIFPLAVNSARADVKSAG	Non-toxin
D-alanyl-D-alanine carboxypeptidase	A9WUR8	–	–	DVAVGEIAPIFPLAV	Non-toxin	–	–
Sugar ABC transporter permease	A9WUD1	–	–	FFFMFAFDGVNIKIF	Non-toxin	AFDGVIN	Non-toxin
Mg²^+^ transport ATPase protein C	A9WQM5	LPEERLRSVQSDSDATP	Non-toxin	EFRLRSVQSDSDATP	Non-toxin	TPGEVRIAAEL	Non-toxin
Mg²^+^ transport ATPase protein C	A9WQM5	AELTAHERDDRQLEAAVSRLSLEPRV	Non-toxin	AAVSRLSLEPRVTSV	Non-toxin	LEAAVSRLSLEPRVTSVRW	Non-toxin
p57	Q9R3F0	SGDNNTYGG	Non-toxin	–	–	–	–
p57	Q9R3F0	–	–	GTVVKVDGSNLFGAS	Non-toxin	GGGTVVKVDG	Non-toxin
p57	Q9R3F0	–	–	FLYLRGSGVYYLFG	Non-toxin	GGSGVYYLF	Non-toxin
Heme-binding periplasmic protein (HmuT)	A9WRJ5	ASHDRGGDVD	Non-toxin	–	–	GGDVQV	Non-toxin
Heme-binding periplasmic protein (HmuT)	A9WRJ5	TSPNGQSVNAE	Non-toxin	–	–	VNAESILALKPSVVIT	Non-toxin
Heme-binding periplasmic protein (HmuT)	A9WRJ5	–	–	KVSAQKLRMAFLYLR	Non-toxin	KLRMAFLYL	Non-toxin
Hemin-binding periplasmic protein (HmuU.1)	A9WRJ2	LLFSTAGQSSAGQA	Non-toxin	IVAPLLFSTAGQSS	Non-toxin	AAFGASAAVAPLLFS	Non-toxin
dnaJ	A9WQT0	EVLSDPQKRQVYDTTNGENTQSGGSGF	Non-toxin	–	–	–	–
dnaJ	A9WQT0	VEMRVNRDAKFDREGDDLHA	Non-toxin	VEMRVNRDAKFDREG	Non-toxin	–	–
Ferric enterobactin transport system	A9WMV7	TVRLGVPSVQLKP	Non-toxin	MTTVRLGVPSVQLKPRAVLISAVLFVLALMAFHVAYGG	Non-toxin	VRLGPVSFQLKPRAVLISAVLFVLVLALMAFHVAYGGTALPYGKVFVALLG	Non-toxin
Type IV secretion system	A9WP76	TLVVVTLWLVKVVRKR	Non-toxin	IGATLVVVTLWLVV	Non-toxin	SSTAAVLIGATLVVVTLWLVVKV	Non-toxin
HSP70 (DnaK)	A9WQR3	EDELLLVFDLGGGTFDVSLLEVG	Non-toxin	KEDELLLVFDLGGGT	Non-toxin	TFDVSLLEV	Non-toxin
HSP70 (DnaK)	A9WQR3	GPLDIHVLDVTF	Non-toxin	DIHVLDVTFANPSVT	Non-toxin	PLDIHVLDVTFANPSVTI	Non-toxin
Heme transport associated protein	A9WRJ6	SMQPVPLTLNVP	Non-toxin	ASSPRISMQPVPLTL	Non-toxin	ISMQPVPLTLNVPTSGQTAVFRVEASASPSQW	Non-toxin
Heme transport associated protein	A9WRJ6	VTTEKAPEPQLPIDPTATSRPGST	Non-toxin	–	–	PEPQLPID	Non-toxin
Heme transport associated protein	A9WRJ6	–	–	LNLTFSNPQLSIDSA	Non-toxin	FSNPQLSID	Non-toxin

Each epitope was analyzed for toxicity using the ToxinPred server. All epitopes included in this study were predicted to be non-toxic.

### Design and *in silico* evaluation of the multiantigenic and multiepitope chimeric protein

Based on structural modeling and molecular docking analyses, antigenic regions showing the most favorable interactions with Atlantic salmon MHC class I and II molecules were selected for construction of the chimeric protein. Priority was given to regions derived from proteins belonging to different functional subsystems, including virulence, secretion systems, iron acquisition and metabolism, chaperones, and carbohydrate transport, to maximize antigenic diversity within a single construct (**Supplementary Table 10**). A total of 12 antigenic regions were selected and sequentially assembled into a single chimeric protein using the flexible amino acid linker GSGSGS (**Table 4**). For each selected region, amino acid sequence, positional information, length, and corresponding HADDOCK docking scores against MHC class I and II alleles were documented.

**Table 4:** Selected antigenic regions for chimeric construct design.

Subsystem	Protein ID (Name)	Antigenic region	Position	# residues	HADDOCK score MHC I	HADDOCK score MHC II
Chaperone	A9WQT0 (DnaJ)	AEEKFKNVSHAYEVLSDPQKRQVYDTTGNENGTQSGGSGF	40–79	40	−85.1	−130.2
	A9WQR3 (DnaK)	KEDELLLVFDLGGGTFDVSLLEV	161–183	23	−73.3	−120.7
Virulence	Q9R3F0 (P57)	GGGTVVKVDGSNLFGAS	164–180	17	−95.8	−124.0
		SGDNN TYG GWF	344–354	11	−84.2	−143.0
	A9WUR8 (DacB)	AALLGQVVRLMTQGPDANVRRAVDG	366–390	25	−84.1	−120.0
	A9WN89 (TpL)	LAGVFTSSSSGCSRSLQTGIDGGSGQKRRDRHDDPACLWHFAANR	96–139	44	−154.6	−116.5
Secretion systems	A9WN91 (TadE)	ITTLKITVRAPMPIIGLIGL	96–115	20	−83.2	−123.2
	A9WP76 (T4SS)	SSTAAVLIGATLVVVTLWLVKVKR	4–30	27	−71.2	−152.0
Iron acquisition and metabolism	A9WRJ5 (HmuT)	KVSAQKLRMAFLYLRGGSGVYYLFG	241–265	25	−122.0	−140.0
	A9WMV7 (FepG)	TVRLGVPSFQLKPRAVLISAVLFVLVLALMAFHVAYGGTALPYGKVFVALLG	3–54	52	−151.5	−151.1
	A9WRJ2 (HmuU)	PGVVGSSGAAFGASAAIVAVPLLFS TAGQSSAGQA AAPSWLIAGFAFLGALAAALLVY	104–162	59	−89.3	−128.1
Sugar import	A9WUD1 (SugA)	TAVTAKLFFFMFAFDGVNIKIFTGQLIWTG	164–193	30	−85.9	−131.8

Twelve antigenic regions from different functional subsystems were selected for chimeric protein design, and their sequences, positions, residue numbers, and HADDOCK docking scores against *Salmo salar* MHC class I (UBA*0201) and MHC class II (DAA*0201–DAB*0201) are shown.

**Figure 1:**
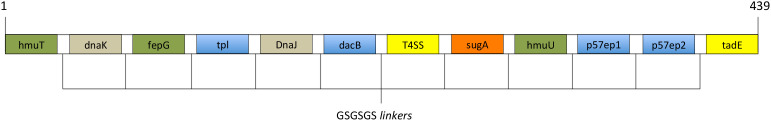
Chimera’s epitope organization. 12 antigenic regions from 5 subsystem proteins were joined by a GSGSGS aminoacidic *linker*. The final construct has 439 aminoacids. Green: Haemin and iron acquisition system; Brown: Chaperons; Blue: Virulence; Yellow: Secretion system: Oranje: Sugar importer.

The designed chimeric protein consists of 439 amino acids and presents a predicted molecular weight of 43.7 kDa and a basic isoelectric point (pI = 9.56). The aliphatic index (87.97) suggests good thermostability, while solubility prediction indicated moderate solubility. Toxicity analysis classified the construct as non-toxic. These physicochemical and structural properties support the suitability of the chimeric protein for further *in silico* and experimental evaluation (**Table 5**; **Supplementary Table 11**). **Figure 1** outlines the chimera structure and epitope organization.

**Table 5:** Physicochemical properties of chimeric proteins predicted using the ProtParam tool.

Property	Value
Number of amino acids	439
Molecular weight	43.7 kDa
Isoelectric point (pI)	9.56
Estimated half-life	1.3 hours (*in vitro*); 3 minutes (*in vivo*)
Aliphatic index	87.97
Solubility	0.43
Toxicity	Non-toxic

No homologous protein models exist for the designed chimera; therefore, *ab initio* protein modeling was performed. The best predicted three-dimensional model was selected based on comparison of its secondary structure with the previously determined epitope structural models and its QMEAN quality score. The selected model was further subjected to refinement using molecular dynamics simulation with NAMD (100 ns), resulting in a refined structure.

**Figure 2:**
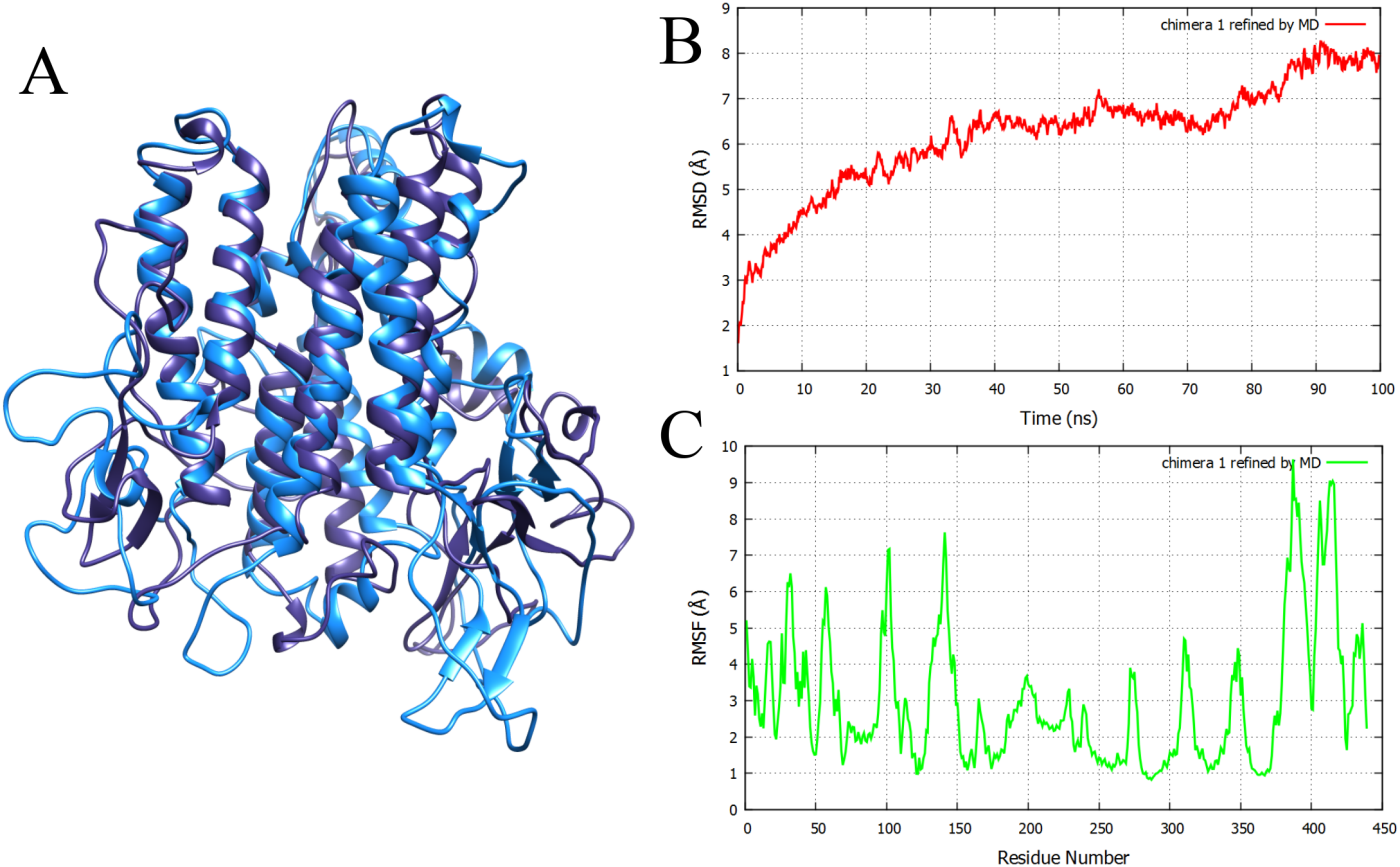
Molecular dynamics of the Chimeric protein. The best model obtained from the Robetta server was used to perform a molecular dynamic with NAMD2. **(A)** Overlapping of model number 3 (dark blue) and the final model obtained from refinement (light blue). **(B)** Root mean-square deviation over the 100ns’s molecular dynamics, noticing a convergence at 85 ns. **(C)** Root means square fluctuation related to the aminoacidic position, showing the highest fluctuation at around the 400 position.

The RMSD profile showed a stabilization trend after ~85 ns, indicating structural convergence during the simulation. RMSF analysis identified localized flexibility, particularly around the ~400 amino acid region. **Figure 2** shows the structural superposition of the chimera before and after refinement, together with the corresponding RMSD and RMSF plots.

**Figure 3:**
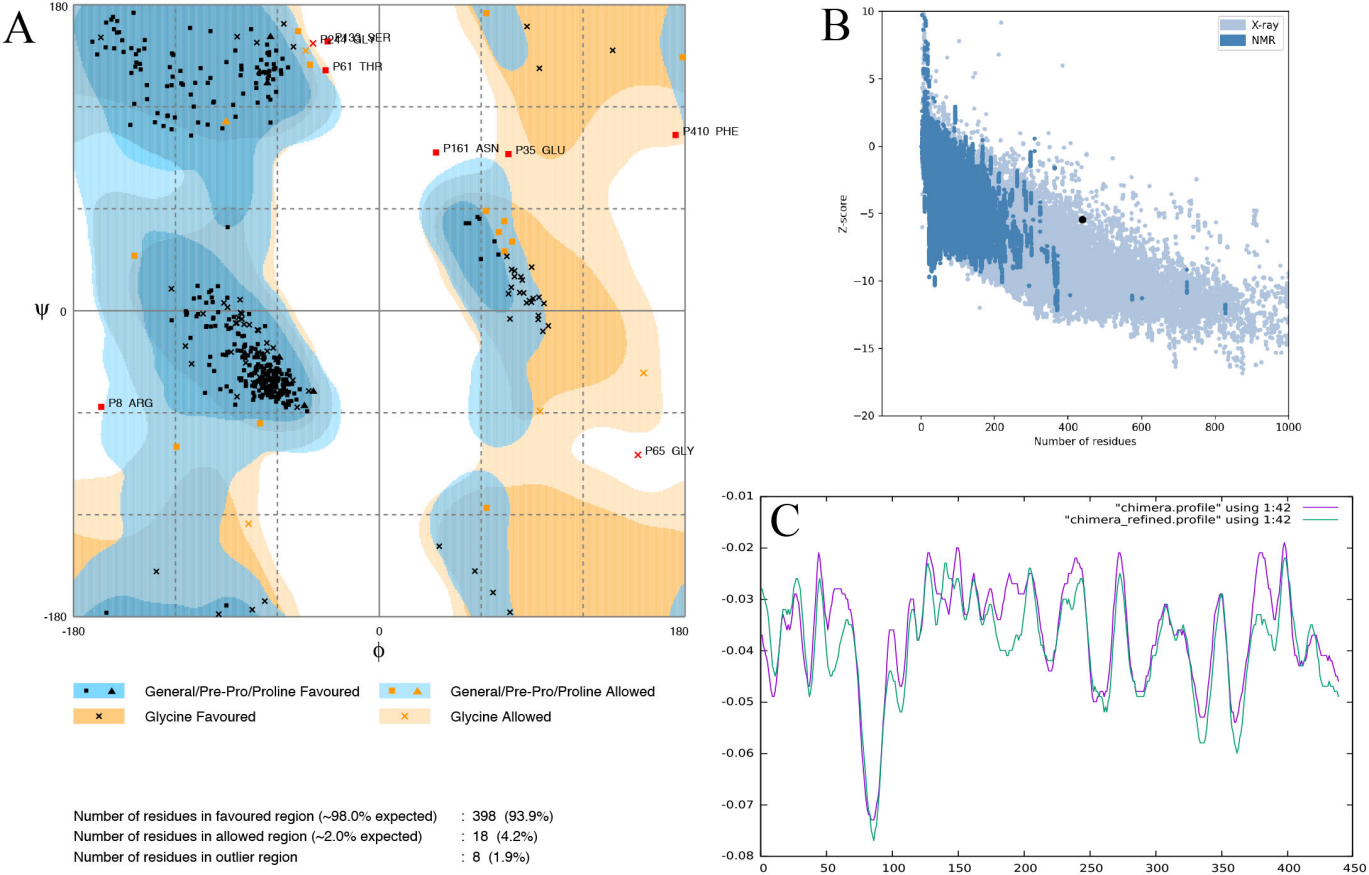
Quality analysis. To validate the final model, a quality analysis was performed comparing both previously and following refinement models. **(A)** Ramachandran plot analysis of refined chimera, after finement the residues located in allowed regions improved from 92.9% to 93.9%. **(B)** ProsaWeb analysis after refinement, both of the structures got a quality tipically seen in solved protein structures. **(C)** DOPE local energy graphic representation before (violet line) and after (green line) refinement.

To validate the refined chimera model, structural quality analyses were performed using Ramachandran plot analysis, ProSA-web, and DOPE energy profiling with the Modeller evaluate script (v9.23). After refinement, the percentage of residues within acceptable regions of the Ramachandran plot improved from 92.9% to 93.9%. Although a detailed distinction between favored and additionally allowed regions was not explicitly quantified, the overall distribution of residues indicates a structurally reliable model. In both cases, the chimera model was within the range of experimentally resolved protein structures according to ProSA-web analysis. DOPE energy profiling showed a decrease in energy values, particularly around the 200 and 400 amino acid positions, when comparing pre- and post-refinement models (**Figure 3**).

**Figure 4:**
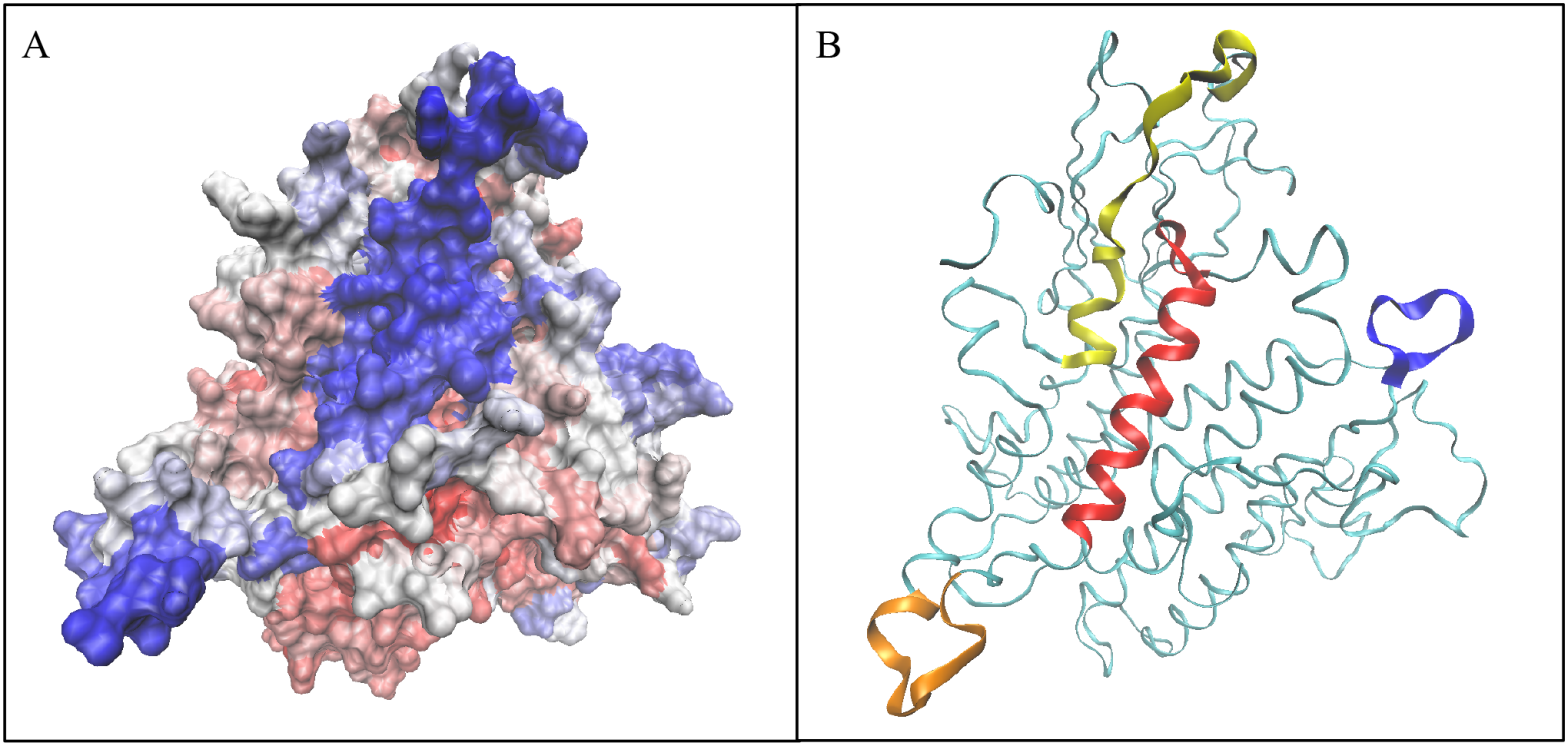
Antigenic prediction on chimera surface. **(A)** Visualization of the most antigenic regions in the refined chimera surface, the residues are colored from red to blue according to the epitopic and antigenic probability is increasing. **(B)** The most antigenic and epitopic regions were colorated in order to the related epitope. Yellow, DacB; Red, Tpl; Orange, DnaK; Blue, HmuU.

EPCES analysis identified the most antigenic regions on the surface of the chimeric protein, corresponding to epitopes derived from DnaK, HmuU, Tpl, and DacB proteins. These proteins are associated with key biological functions, including protein folding, iron acquisition, and cell wall remodeling. The surface localization of these epitopes within the chimeric construct supports their accessibility for potential immune recognition (**Figure 4**).

The physicochemical, solubility, and toxicity properties are summarized in **Table 5**, confirming a non-toxic profile and suitable physicochemical characteristics for the designed construct.

## Discussion

Bacterial kidney disease (BKD), caused by the Gram-positive intracellular pathogen *R. salmoninarum*, remains one of the most persistent and challenging bacterial diseases affecting salmon aquaculture. Its chronic progression, ability to spread through both horizontal and vertical transmission routes, and capacity to evade host immune responses have significantly limited the effectiveness of current control strategies (Bruno, 2015; Evelyn et al., 1986). In Chile, the lack of a fully effective vaccine has resulted in a continued reliance on antimicrobial treatments, contributing to environmental contamination and the emergence of antimicrobial resistance, including the documented transfer of resistance determinants from aquaculture-associated bacteria to human pathogens (Aedo et al., 2014; Tomova et al., 2015, Tomova et al., 2018).

In this context, the application of integrative *in silico* approaches represents a rational and cost-effective alternative to traditional vaccine development. Reverse vaccinology and immunoinformatics enable the systematic exploration of pathogen genomes and proteomes to identify immunogenic targets without the need for extensive *in vitro* or *in vivo* screening (Rappuoli, 2000; Seib et al., 2012). These methodologies have been successfully applied to the design of vaccine candidates against a wide range of bacterial pathogens of both medical and veterinary relevance, demonstrating their versatility and predictive power (Doytchinova and Flower, 2007; He and Xiang, 2010).

The present study applied an integrative framework combining comparative genomics, proteomic annotation, antigenicity prediction, epitope mapping, structural modeling, and molecular docking to identify conserved immunogenic targets in *R. salmoninarum*. Genomic and proteomic analyses revealed a high degree of conservation among the available strains, supporting the selection of shared antigenic determinants that could potentially provide broad coverage across different isolates. Similar levels of conservation have been reported previously for *R. salmoninarum*, reinforcing the suitability of conserved proteins and subsystems as vaccine targets (Bethke et al., 2018).

Protein selection was guided not only by antigenicity predictions but also by functional relevance and subcellular localization. Proteins associated with virulence, secretion systems, iron acquisition, and surface exposure were prioritized due to their established roles in bacterial pathogenicity and host–pathogen interactions. In addition, chaperones and ribosomal proteins were included based on growing evidence supporting their immunomodulatory properties and capacity to elicit protective immune responses in both bacterial and parasitic infections (Neckers and Tatu, 2008; Johnson et al., 2017). This multi-subsystem selection strategy aligns with current vaccine design paradigms that emphasize antigenic diversity to reduce immune escape.

Epitope prediction and selection focused on identifying antigenic regions capable of stimulating B- and T-cell responses while minimizing the risk of host cross-reactivity. In teleost fish, adaptive immune memory has been primarily associated with B lymphocytes and antibody production, highlighting the importance of selecting accessible and highly antigenic epitopes (Morrison and Nowak, 2002; Ye et al., 2013). The exclusion of epitopes conserved in salmonid proteomes further strengthens the safety profile of the proposed construct by reducing the likelihood of autoimmunity, a key consideration in rational vaccine design.

Structural modeling and molecular docking analyses provided additional support for epitope selection by evaluating the interaction of antigenic regions with Atlantic salmon MHC class I and II molecules. The relevance of MHC–peptide binding in fish immunity has been increasingly recognized, with specific alleles being associated with enhanced resistance to infectious diseases (Kjøglum et al., 2006; Grimholt, 2016). The identification of antigenic regions with favorable predicted interactions with salmonid MHC molecules suggests that the selected epitopes may be efficiently presented to the adaptive immune system, thereby enhancing their immunogenic potential. Despite the emphasis placed on B-cell and CD4+ T helper epitopes in the present study, it is important to acknowledge that adaptive immunity in salmonids also involves CD8+ cytotoxic T lymphocytes (CTLs), supported by the presence of functional MHC class I molecules and associated antigen presentation pathways (Grimholt, 2016). CTL responses have been experimentally demonstrated in salmonids, particularly in the context of viral infections, and may also play a relevant role in defense against intracellular bacterial pathogens such as *R. salmoninarum*. Although MHC class I molecules were included in the docking analyses performed in this study, the design strategy did not explicitly prioritize CD8+ T cell epitopes. This aspect highlights an important direction for future research, where the integration of CTL-targeted epitopes could further enhance the protective potential of vaccine candidates.

The final chimeric protein integrated antigenic regions derived from multiple functional subsystems of *R. salmoninarum*, resulting in a multiantigenic and multiepitope construct. The use of a flexible GSGSGS linker was intended to preserve epitope independence and maintain conformational accessibility within the chimeric construct. Given the intracellular nature of *R. salmoninarum*, the design is consistent with antigen processing pathways that may lead to peptide generation and presentation via MHC class I. However, the precise processing and presentation of the construct require experimental validation. Such designs have been shown to enhance immune recognition and breadth of response compared to single-antigen vaccines, particularly against pathogens with complex infection strategies (Skwarczynski and Toth, 2016; Negahdaripour et al., 2018). *In silico* evaluation of physicochemical properties, solubility, antigenicity, and toxicity indicated that the construct possesses favorable characteristics for further development, supporting its suitability for downstream experimental validation. The present construct does not include a genetically fused adjuvant sequence, which is commonly used in multi-epitope vaccine design to enhance immunogenicity. While this represents a limitation, the current design focused on the identification and structural integration of highly antigenic regions. In practical applications, the chimeric protein could be formulated with conventional adjuvants, such as oil-based emulsions widely used in fish vaccines, or alternatively optimized by incorporating molecular adjuvants (e.g., TLR agonists) in future iterations. This represents an important direction for further development and experimental validation.

Finally, the predicted half-life of the chimeric protein, as estimated by the ProtParam tool (**Table 5**), suggests a relatively short intrinsic stability. Although such predictions are based solely on primary sequence features and do not account for structural conformation, formulation, or delivery systems, they may indicate a potential limitation for *in vivo* applications. In vaccine development, however, protein stability can be significantly enhanced through the use of adjuvants, carrier systems, or fusion strategies that prolong antigen persistence and improve immune recognition. Therefore, while the predicted half-life should be considered cautiously, it highlights the importance of future experimental work aimed at optimizing protein stability and delivery.

Compared to existing vaccine strategies against BKD, such as the commercial Renogen^®^ vaccine or recombinant approaches based on the p57 antigen, the multi-epitope chimeric construct proposed in this study offers broader antigenic coverage. While p57-based strategies focus on a single antigen, the present design integrates epitopes from multiple functional subsystems, potentially reducing the risk of immune evasion and improving overall immune response diversity.

While the present study is limited to computational and bioinformatic analyses, it provides a comprehensive and rational framework for develop vaccine candidates against BKD. Experimental validation, including expression, purification, and immunogenicity testing in relevant salmonid models, will be required to confirm the protective efficacy of the proposed construct. Nevertheless, this work demonstrates the potential of integrative *in silico* methodologies to accelerate vaccine development and offers a promising strategy to reduce antibiotic dependence and improve disease control in salmon aquaculture.

## Conclusions

This study presents an integrative *in silico* strategy for the rational design of a multiantigenic and multiepitope chimeric protein as a vaccine candidate against *Renibacterium salmoninarum*, the etiological agent of bacterial kidney disease in salmonids. By combining comparative genomics, proteomic annotation, antigenicity prediction, epitope mapping, structural modeling, and molecular docking, conserved immunogenic targets from diverse functional subsystems of the pathogen were systematically identified and evaluated.

The proposed chimeric construct integrates antigenic regions derived from proteins associated with virulence, secretion systems, iron acquisition, chaperones, and carbohydrate transport, and exhibits favorable predicted physicochemical properties, antigenicity, solubility, and safety profiles. Importantly, selected antigenic regions showed no conservation in salmonid host proteomes and demonstrated predicted interactions with Atlantic salmon MHC class I and II molecules, supporting their potential relevance for adaptive immune recognition.

Although experimental validation is required to confirm immunogenicity and protective efficacy, the results of this work provide a solid conceptual and methodological foundation for the development of novel vaccine strategies against BKD. This approach may contribute to reducing antibiotic dependence and improving disease control in salmon aquaculture.

## Data Availability

The original contributions presented in the study are included in the article/**Supplementary Material**. Further inquiries can be directed to the corresponding author.
