# Supplementary material for: In silico design of a multiantigenic and multiepitope chimeric protein as a vaccine candidate against Renibacterium salmoninarum
Source: Front Cell Infect Microbiol. 2026 Jun 5;16:1814020. doi: 10.3389/fcimb.2026.1814020 (PMC13279606; doi:10.3389/fcimb.2026.1814020)
Supplement: Supplementary file 1 [file Table1.docx]

**Supplementary data**


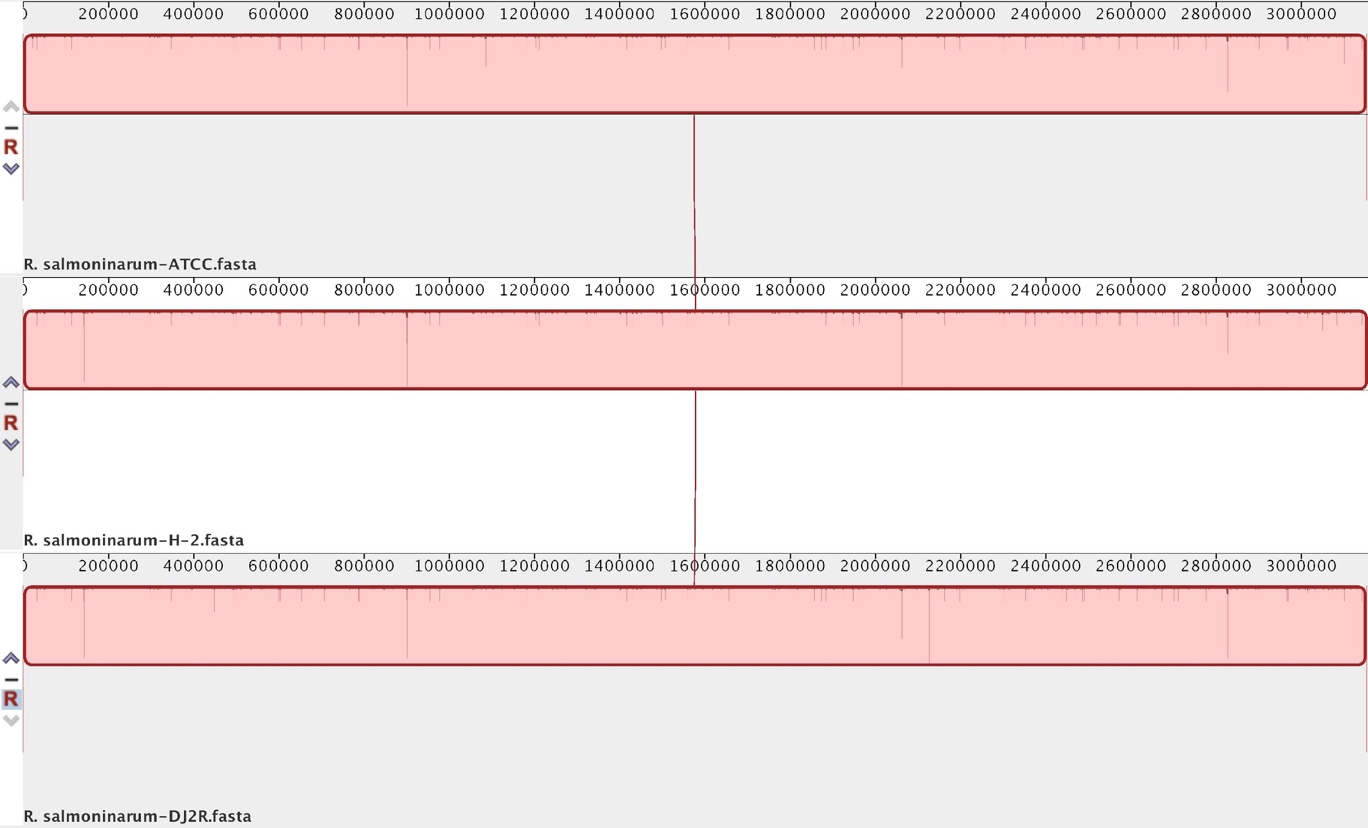


**Figure S1. Whole-genome alignment of** R. salmoninarum **strains using Mauve 2.3.1.**Whole-genome alignment of Renibacterium salmoninarum strains ATCC 33209 (GenBank accession number: CP000910), H-2 (GenBank accession number: CP029236), and DJ2R (GenBank accession number: CP029237) was performed using the Mauve software. A single collinear block spanning the entire genome was observed, indicating a high level of genomic conservation among the analyzed strains. Localized variation regions are indicated by red lines.

| **Strain** | **Genome length (bp)** | **Subsystems** | **GC content (%)** | **Coding sequences** |
| --- | --- | --- | --- | --- |
| ATCC 33209 | 3,155,250 | 260 | 56.3 | 3,715 |
| H-2 | 3,155,332 | 260 | 56.3 | 3,719 |
| DJ2R | 3,155,228 | 260 | 56.3 | 3,716 |

**Table S1. Proteomic annotation using the RAST server (version 4).**The three strains show high similarity in the number of protein subsystems (260), GC content (56.3%), and coding sequences.

The three *Renibacterium salmoninarum* strains were subjected to comprehensive proteomic annotation using the RAST server. The results show a high level of similarity among strains in terms of genome length, number of identified protein subsystems, GC content, and number of coding sequences.

| **Subsystem** | **Protein name** | **Accession** |
| --- | --- | --- |
| **Type II secretion system** | Type II secretion system type E | A9WN85 |
|  | Type II secretion system type E | A9WN86 |
|  | Flp pilus assembly protein TadB | A9WN87 |
|  | Type II secretion system protein | A9WN88 |
|  | Tyrosine phenol-lyase | A9WN89 |
|  | Hypothetical protein | A9WN90 |
|  | TadE family protein | A9WN91 |
|  | Hypothetical protein | A9WPT5 |
| **Virulence** | Multidrug resistance efflux pump | A9WVK7 |
|  | SSU ribosomal protein S12p | A9WSW8 |
|  | SSU ribosomal protein S7p | A9WSW7 |
|  | Translocation elongation factor G | A9WSW6 |
|  | Translocation elongation factor Tu | A9WSW5 |
|  | LSU ribosomal protein L20 | A9WQB1 |
|  | LSU ribosomal protein L35 | A9WQB0 |
|  | Translation initiation factor 3 | A9WQA9 |
| **Cell wall and capsule** | Peptidoglycan lipid flippase MurJ | A9WVN5 |
|  | D-alanyl-D-alanine carboxypeptidase | A9WMF6 |
|  | D-alanyl-D-alanine carboxypeptidase | A9WUR8 |
|  | Phosphoglucosamine mutase | A9WMF8 |
|  | N-acetylglucosamine-1-phosphate uridyltransferase | A9WU39 |
|  | UDP-N-acetylglucosamine 1-carboxyvinyltransferase | A9WNV9 |
|  | UDP-N-acetylglucosamine 1-carboxyvinyltransferase | A9WMG8 |
| **Iron acquisition and metabolism** | Phosphatidylinositol phosphate synthase | A9WSF5 |
|  | Ferric enterobactin transport system, periplasmic component | A9WMV9 |
|  | ABC-type Fe³⁺-siderophore transport system, permease component | A9WMV8 |
|  | Transport system permease protein | A9WMV7 |
|  | ABC-type Fe³⁺-siderophore transport system, ATPase component | A9WMV6 |
|  | Iron transport protein | A9WMT1 |
|  | Transport system for ferric enterobactin (siderophore) | A9WV36 |
|  | Putative transporter | A9WRJ2 |
|  | Putative transporter | A9WRJ3 |
|  | Heme ABC transporter, ATPase component HmuV | A9WRJ4 |
|  | Heme ABC transporter, cell surface heme and hemoprotein receptor HmuT | A9WRJ5 |
|  | Conserved secreted protein | A9WRJ6 |

**Table S2**. Proteins identified by RAST annotation according to functional subsystems.

**Table S3. Proteomic annotation retrieved from the UniProt database.**Proteins mainly associated with virulence, including p57, p22, and chaperones, as well as proteins related to the type IV secretion system, were selected.

| **Protein name** | **UniProt accession** |
| --- | --- |
| p57 | Q9R3F0 |
| p22 | Q9R4V6 |
| HSP70 (DnaK) | A9WQR3 |
| HSP40 (DnaJ) | A9WQT0 |
| Type IV secretion system protein | A9WP76 |
| Type IV secretion system protein | A9WP78 |

| **Protein name** | **UniProt accession** | **Match** | **Identity (%)** | **Species** |
| --- | --- | --- | --- | --- |
| Chaperone GroEL | A9WMA0 | P9WPE7 | 84.4 | *Mycobacterium tuberculosis* H37Rv |
| Sigma factor | A9WS22 | A0QW02 | 75.7 | *Mycobacterium smegmatis* str. MC2 155 |
| DNA-binding response regulator | A9WLY0 | J9WHZ5 | 75.7 | *Mycobacterium indicus pranii* MTCC 9506 |
| Aspartate 1-decarboxylase | A9WT19 | F5YS92 | 70.5 | *Mycobacterium* sp. JDM601 |
| Superoxide dismutase | A9WT65 | P53647 | 69.3 | *Mycobacterium avium* subsp. *paratuberculosis* K-10 |
| 3-isopropylmalate dehydratase small subunit | A9WMG9 | B1MDQ0 | 68.8 | *Mycobacterium abscessus* ATCC 19977 |
| UDP-glucose 4-epimerase | A9WN76 | Q4L9H4 | 66.8 | *Staphylococcus haemolyticus* JCSC1435 |
| Protein ligase | A9WSH9 | B1MAJ3 | 66.7 | *Mycobacterium abscessus* ATCC 19977 |
| Sugar ABC transporter permease | A9WUD1 | A0A1R3XXS7 | 66.2 | *Mycobacterium bovis* AF2122/97 |
| Type I glutamate–ammonia ligase | A9WS44 | F5Z0W7 | 65.9 | *Mycobacterium* sp. JDM601 |
| Sigma factor RpoE | A9WPK7 | G0THJ9 | 65.6 | *Mycobacterium canettii* CIPT 140010059 |
| Elongation factor Tu | A9WSW5 | Q4A597 | 70.0 | *Mycoplasma synoviae* 53 |
| XRE family transcriptional regulator | A9WLG9 | F5Z0R9 | 69.0 | *Mycobacterium* sp. JDM601 |
| Mg²⁺ transport ATPase protein C | A9WQM5 | R4UQM0 | 65.0 | *Mycobacterium abscessus* subsp. *bolletii* 50594 |
| LSU ribosomal protein L10p (P0) | A9WNU8 | P9WHE7 | 49.0 | *Mycobacterium tuberculosis* (strain ATCC 25618 / H37Rv) |
| SSU ribosomal protein S1p | A9WT92 | P9WH43 | 78.0 | *Mycobacterium tuberculosis* (strain ATCC 25618 / H37Rv) |
| ABC transporter | A9WQ94 | A0A1I9ZL11_9BACL | 80.2 | *Paenibacillus* sp. LC231 |

**Table S4.** Homology analysis of selected proteins against related bacterial species.

**Table S5. VaxiJen scores for the three groups of selected proteins.**
A total of 27 proteins were predicted as “probably antigenic” based on VaxiJen scores above 0.5.

| **Group** | **Protein name** | **UniProt accession** | **VaxiJen score** |
| --- | --- | --- | --- |
| **RAST server** | Type II secretion system type E | A9WN85 | 0.2810 |
|  | Type II secretion system type E | A9WN86 | 0.3965 |
|  | Flp pilus assembly protein TadB | A9WN87 | 0.4492 |
|  | Type II secretion system protein | A9WN88 | 0.4607 |
|  | Tyrosine phenol-lyase | A9WN89 | 0.5116 |
|  | Hypothetical protein | A9WN90 | 0.4101 |
|  | TadE family protein | A9WN91 | 0.5819 |
|  | Hypothetical protein | A9WPT5 | 0.3401 |
|  | Hypothetical protein | A9WPT5 | 0.7415 |
|  | Multidrug resistance efflux pump | A9WVK7 | 0.4534 |
|  | SSU ribosomal protein S12p | A9WSW8 | 0.8617 |
|  | SSU ribosomal protein S7p | A9WSW7 | 0.7114 |
|  | Translocation elongation factor G | A9WSW6 | 0.6024 |
|  | Translocation elongation factor Tu | A9WSW5 | 0.4074 |
|  | LSU ribosomal protein L20 | A9WQB1 | 0.3285 |
|  | LSU ribosomal protein L35 | A9WQB0 | 0.7438 |
|  | Translation initiation factor 3 | A9WQA9 | 0.7016 |
|  | Peptidoglycan lipid flippase MurJ | A9WVN5 | 0.4140 |
|  | D-alanyl-D-alanine carboxypeptidase | A9WMF6 | 0.4428 |
|  | D-alanyl-D-alanine carboxypeptidase | A9WUR8 | 0.5142 |
|  | Phosphoglucosamine mutase | A9WMF8 | 0.5611 |
|  | N-acetylglucosamine-1-phosphate uridyltransferase | A9WU39 | 0.6475 |
|  | UDP-N-acetylglucosamine 1-carboxyvinyltransferase | A9WNV9 | 0.5842 |
|  | UDP-N-acetylglucosamine 1-carboxyvinyltransferase | A9WMG8 | 0.4862 |
|  | Phosphatidylinositol phosphate synthase | A9WSF5 | 0.4548 |
|  | Ferric enterobactin transport system | A9WMV9 | 0.4381 |
|  | ABC-type Fe³⁺-siderophore transport | A9WMV8 | 0.3398 |
|  | Transport system permease protein | A9WMV7 | 0.5518 |
|  | ABC-type Fe³⁺-siderophore transport | A9WMV6 | 0.3712 |
|  | Iron transport protein | A9WMT1 | 0.4187 |
|  | Transport system for ferric enterobactin | A9WV36 | 0.4409 |
|  | Putative transporter | A9WRJ2 | 0.6003 |
|  | Putative transporter | A9WRJ3 | 0.3280 |
|  | Heme ABC transporter, ATPase component | A9WRJ4 | 0.3135 |
|  | Heme ABC transporter, cell surface heme and hemoprotein receptor | A9WRJ5 | 0.7008 |
|  | Conserved secreted protein | A9WRJ6 | 0.5903 |
| **UniProt** | p57 | Q9R3F0 | 0.6465 |
|  | p22 | Q9R4V6 | 0.4049 |
|  | HSP70 (DnaK) | A9WQR3 | 0.6346 |
|  | HSP40 (DnaJ) | A9WQT0 | 0.8160 |
|  | Type IV secretion system protein | A9WP76 | 0.5557 |
|  | Type IV secretion system protein | A9WP78 | 0.4196 |
|  | Molecular chaperone GroEL | A9WMA0 | 0.4738 |
|  | RNA polymerase sigma factor | A9WS22 | 0.4415 |
|  | DNA-binding response regulator | A9WLY0 | 0.5440 |
|  | Aspartate 1-decarboxylase | A9WT19 | 0.3788 |
|  | Superoxide dismutase | A9WT65 | 0.4869 |
| **Virulence-associated homologous proteins** | 3-isopropylmalate dehydratase small subunit | A9WMG9 | 0.5676 |
|  | UDP-glucose 4-epimerase | A9WN76 | 0.3317 |
|  | Pup–protein ligase | A9WSH9 | 0.4820 |
|  | Sugar ABC transporter permease | A9WUD1 | 0.5533 |
|  | Type I glutamate–ammonia ligase | A9WS44 | 0.4736 |
|  | RNA polymerase sigma factor RpoE | A9WPK7 | 0.5156 |
|  | Elongation factor Tu | A9WSW5 | 0.4074 |
|  | XRE family transcriptional regulator | A9WLG9 | 0.5791 |
|  | Mg²⁺ transport ATPase protein C | A9WQM5 | 0.5913 |
|  | LSU ribosomal protein L10p (P0) | A9WNU8 | 0.4008 |
|  | SSU ribosomal protein S1p | A9WT92 | 0.4498 |
|  | ABC transporter | A9WQ94 | 0.6140 |

**Table S6. Subcellular localization prediction of selected proteins.**
The 27 proteins were analyzed using three different prediction servers: Protter, Gpos-mPLoc, and PSORTb. Proteins predicted as membrane-associated, secreted, chaperones, and ribosomal proteins were considered for subsequent analyses.

| **Group** | **UniProt accession** | **Protter** | **Gpos-mPLoc** | **PSORTb** |
| --- | --- | --- | --- | --- |
| **RAST-derived proteins** | A9WN89 | Intracellular | Cytoplasm / extracellular | Cytoplasm / extracellular |
|  | A9WN91 | Membrane | Extracellular | Membrane |
|  | A9WPT5 | Intracellular | Membrane / extracellular | Unknown |
|  | A9WSW8 | Intracellular | Cytoplasm | Cytoplasm |
|  | A9WSW7 | Intracellular | Cytoplasm | Cytoplasm |
|  | A9WSW6 | Intracellular | Cytoplasm | Cytoplasm |
|  | A9WQB0 | Intracellular | Cytoplasm | Cytoplasm |
|  | A9WQA9 | Intracellular | Cytoplasm | Cytoplasm |
|  | A9WUR8 | Extracellular | Extracellular | Extracellular |
|  | A9WMF8 | Intracellular | Cytoplasm | Unknown |
|  | A9WU39 | Intracellular | Cytoplasm | Cytoplasm |
|  | A9WNV9 | Intracellular | Cytoplasm | Cytoplasm |
|  | A9WMV7 | Membrane | Membrane | Membrane |
|  | A9WRJ2 | Membrane | Membrane | Membrane |
|  | A9WRJ5 | Membrane | Membrane | Unknown |
|  | A9WRJ6 | Membrane | Extracellular | Extracellular |
| **UniProt-derived proteins** | Q9R3F0 | Extracellular | Extracellular | Unknown |
|  | A9WQR3 | Intracellular | Cytoplasm | Cytoplasm |
|  | A9WQT0 | Intracellular | Cytoplasm | Cytoplasm |
|  | A9WP76 | Membrane | Extracellular | Membrane |
| **Virulence-associated homologous proteins** | A9WLY0 | Intracellular | Cytoplasm | Cytoplasm |
|  | A9WMG9 | Intracellular | Cytoplasm | Cytoplasm |
|  | A9WUD1 | Membrane | Membrane | Membrane |
|  | A9WPK7 | Intracellular | Membrane | Intracellular |
|  | A9WLG9 | Intracellular | Cytoplasm | Cytoplasm |
|  | A9WQM5 | Membrane | Membrane | Membrane |
|  | A9WQ94 | Intracellular | Membrane | Membrane |

**Table S7.** Epitope prediction.
The 17 selected proteins were subjected to epitope prediction using the IMED, IEDB, and BcePred servers. For each protein, epitopes with shared or similar predictions among servers, as well as those with the highest scores (according to the algorithm used by each server), were primarily annotated. A total of 154 epitopes corresponding to 69 antigenic regions were finally identified.

**Table S8:** **Proteomic alignment using BLAST.**
Each protein associated with the selected epitope regions was subjected to a homology search against the BLAST database restricted to the teleost species included in this study (*Oncorhynchus kisutch*, *Salmo salar*, and *Oncorhynchus mykiss*). Proteins identified in host species were aligned against the selected epitopes using CLC Genomics Workbench version 6.9.1. None of the antigenic regions were conserved in the host proteins (results not shown), thereby ruling out the potential for cross-reactive immunity.

| **Protein name** | **Best match (GenPept)** | **identity %** | **Coverage %** | **Organism** |
| --- | --- | --- | --- | --- |
| Tyrosine phenol-lyase | Not found | – | – | – |
| TadE family protein | Not found | – | – | – |
| Hypothetical protein | Not found | – | – | – |
| SSU ribosomal protein S12p | XP_021474234 | 46.85 | 89 | *Oncorhynchus mykiss* |
| D-alanyl-D-alanine carboxypeptidase | Not found | – | – | – |
| Sugar ABC transporter permease (sugA) | Not found | – | – | – |
| Mg(2+) transport ATPase protein C | XP_021454943.1 | 41.18 | 14 | *Oncorhynchus mykiss* |
| p57 | XP_021425210.1 | 31.55 | 30 | *Oncorhynchus mykiss* |
| Hemin-binding periplasmic protein (HmuT) | Not found | – | – | – |
| Hemin-binding periplasmic protein (HmuU.1) | Not found | – | – | – |
| dnaJ | XP_014058215.1 | 33.71 | 91 | *Salmo salar* |
| Ferric enterobactin transport system | Not found | – | – | – |
| Type IV secretion system | Not found | – | – | – |
| HSP70 (DnaK) | XP_021416481.1 | 50.33 | 93 | *Oncorhynchus mykiss* |
| Heme transport associated protein | XP_020346140.1 | 26.61 | 8 | *Oncorhynchus kisutch* |

**Table S9: Model quality assessment.**Each model generated using the three prediction servers and Modeller was evaluated using the QMEAN and RAMPAGE (Ramachandran plot analysis) servers. The table reports the global QMEAN scores (QMEANDisCo). Entries marked with a dash (–) correspond to membrane proteins, for which QMEANBrane does not provide a global score. The percentage of residues located in favorable regions, as derived from Ramachandran plots, is also shown. Highlighted models indicate those with the best QMEAN scores and were therefore selected for subsequent three-dimensional structural definition of proteins and epitopes.

| **Protein ID** | **Phyre2 QMEAN score** | **% residues in favorable regions** | **I-TASSER QMEAN score** | **% residues in favorable regions** | **RaptorX QMEAN score** | **% residues in favorable regions** | **Modeller QMEAN score** | **% residues in favorable regions** |
| --- | --- | --- | --- | --- | --- | --- | --- | --- |
| Q9R3F0 | 0.20 | 66.4 | 0.27 | 66.7 | 0.38 | 90.2 | 0.38 | 90.2 |
| A9WQT0 | 0.32 | 74.7 | 0.51 | 72.3 | 0.40 | 95.7 | 0.40 | 95.7 |
| A9WQR3 | 0.22 | 64.6 | 0.71 | 86.3 | 0.74 | 98.1 | 0.74 | 98.1 |
| A9WP76 | 0.26 | 83.4 | 0.29 | 55.4 | 0.29 | 91.7 | 0.29 | 91.7 |
| A9WQM5 | – | 81.2 | – | 65.8 | – | 94.0 | – | 94.0 |
| A9WUD1 | – | 89.8 | – | 79.7 | – | – | – | – |
| A9WN89 | 0.34 | 87.4 | 0.48 | 74.8 | 0.40 | 93.1 | 0.40 | 93.1 |
| A9WN91 | – | 79.5 | – | 54.3 | – | 94.5 | – | 94.5 |
| A9WPT5 | 0.24 | 74.0 | 0.40 | 55.0 | 0.46 | 90.0 | 0.46 | 90.0 |
| A9WSW8 | 0.44 | 77.9 | 0.69 | 81.1 | 0.68 | 95.1 | 0.68 | 95.1 |
| A9WUR8 | 0.55 | 90.7 | 0.55 | 74.4 | 0.57 | 89.8 | 0.57 | 89.8 |
| A9WRJ5 | 0.57 | 92.4 | 0.63 | 75.9 | 0.72 | 94.3 | 0.72 | 94.3 |
| A9WMV7 | – | 95.7 | – | 90.8 | – | 95.1 | – | 95.1 |
| A9WRJ6 | 0.12 | 95.5 | 0.28 | 63.1 | 0.43 | 90.8 | 0.43 | 90.8 |
| A9WRJ2 | – | 89.5 | – | 84.5 | – | 92.0 | – | 92.0 |

| **Subsystem** | **Protein** | **Antigenic region** | **Position** | **HADDOCK score MHC I** | **HADDOCK score MHC II** |
| --- | --- | --- | --- | --- | --- |
| **Chaperones** | A9WQT0 | AEEKFKNVSHAYEVLSDPQKRQVYDTTNGENTQSGGSGF | 40–79 | −85.1 ± 4.0 | −130.2 ± 10.9 |
|  |  | LFVEMRVNRDAKFDREGDDLHAIVSVPMTAAAL | 244–276 | −97.3 ± 15.9 | −113.3 ± 10.7 |
|  | A9WQR3 | KEDELLLVFDLGGGTFDVSLLEV | 161–183 | −73.3 ± 5.7 | −120.7 ± 6.5 |
| **Virulence** | Q9R3F0 | GGGTVVKVDGSNLFGAS | 164–180 | −95.8 ± 6.5 | −124.0 ± 5.1 |
|  |  | SGDNN TYG GWF | 344–354 | −84.2 ± 4.5 | −143.0 ± 9.1 |
|  | A9WUR8 | DPGDVAVGEIAPIFPLAVNSARADVKSAG | 213–242 | −104.3 ± 2.8 | −117.8 ± 11.7 |
|  |  | AALLGQVVRLMTQGPDANVRRAVDG | 366–390 | −84.1 ± 6.7 | −120.0 ± 26.8 |
|  | A9WN89 | LAGVFTSSSSGCSRSLQTGIDGGSGQKRRDRHDDPACLWHFAANR | 96–139 | −154.6 ± 17.3 | −116.5 ± 7.7 |
| **Secretion systems** | A9WPT5 | RSATVTLGAIVHPPINFLIPAGISASTSA | 65–96 | −73.8 ± 9.0 | −111.7 ± 2.9 |
|  | A9WN91 | ITTLKITVRAPMPIIGLIGL | 96–115 | −83.2 ± 4.7 | −123.2 ± 2.4 |
|  | A9WP76 | SSTAAVLIGATLVVVTLWLVKVKR | 4–30 | −71.2 ± 17.5 | −152.0 ± 8.4 |
| **Ribosomal proteins** | A9WSW8 | INQLVRKGRSPKVAKTKAPALKNPMRRGVCTVRYTTTPKPKPNSALRKVARRVL | 4–57 | −98.9 ± 17.5 | −39.3 ± 0.0 |
| **Magnesium acquisition** | A9WQM5 | EDAEAHLRTLIHAVTLPERLRSVQSDSDATPGEVRIAAELTAHERDDRQLEAAVSRLSLEPRVTSVRWTI | 158–229 | −28.8 ± 15.9 | −109.6 ± 33.8 |
| **Iron acquisition and metabolism** | A9WRJ5 | ASVASHDRGGDVDVQV | 84–99 | −70.0 ± 8.1 | −107.6 ± 7.7 |
|  |  | TSPNGQSVNAESILALKPSVVIT | 146–168 | −75.2 ± 7.2 | −103.7 ± 3.4 |
|  |  | KVSAQKLRMAFLYLRGGSGVYYLFG | 241–265 | −122.0 ± 7.2 | −140.0 ± 7.2 |
|  | A9WMV7 | TVRLGVPSFQLKPRAVLISAVLFVLVLALMAFHVAYGGTALPYGKVFVALLG | 3–54 | −151.5 ± 11.0 | −151.1 ± 3.3 |
|  | A9WRJ6 | GPLDIHVLDVTFANPSVTI | 148–166 | −55.7 ± 13.1 | −119.9 ± 4.7 |
|  |  | SSPRISMOPVPLTLNVPTGSQTQVAFRVEASASPSQVW | 636–677 | −71.9 ± 3.4 | −122.5 ± 9.8 |
|  |  | VTTEKAPEPQLPIDPTATSRPGST | 731–754 | −73.7 ± 5.2 | −100.3 ± 4.0 |
|  |  | LNLTFSNPQLSIDSA | 826–840 | −76.9 ± 3.6 | −107.2 ± 4.8 |
|  | A9WRJ2 | PGVVGSSGAAFGASAAIVAVPLLFS TAGQSSAGQA AAPSWLIAGFAFLGALAAALLLVY | 104–162 | −89.3 ± 12.7 | −128.1 ± 12.5 |
| **Sugar import** | A9WUD1 | TAVTAKLFFFMFAFDGVNIKIFTGQLIWTG | 164–193 | −85.9 ± 11.1 | −131.8 ± 4.9 |

**Table S10:** **Selected antigenic regions and HADDOCK docking scores against MHC molecules.**
Selected antigenic regions were evaluated by molecular docking against MHC class I and class II molecules using HADDOCK. Docking scores (mean ± SD) are shown for each antigenic region and were used as a complementary criterion for epitope selection.
